# Anticipation Avoids Adversity: Anesthetic Management of a Case of Facioscapulohumeral Dystrophy (FSHD)

**DOI:** 10.7759/cureus.34442

**Published:** 2023-01-31

**Authors:** Seyed Ahamed, Ramji Swaminathan

**Affiliations:** 1 Anaesthesiology, Amrith Hospital, Chennai, IND; 2 Anesthesiology, NMC Specialty Hospital, Abu Dhabi, ARE

**Keywords:** muscular dystrophies, train of four, neuromuscular monitoring, anaesthesia, fascioscapulohumeral dystrophy

## Abstract

Patients with muscular dystrophies, especially those pauci-symptomatic presenting for surgery pose a complex problem for the anesthesiologist in preparing, optimizing and performing anesthesia. A myriad of complications including cardiac, respiratory, rhabdomyolysis, hyperkalemia, increased sensitivity to muscle relaxants etc., influence the anesthetic technique and recovery. Preoperative identification and appropriate choice of anesthesia technique can prevent most of the adverse events during anesthesia. We present a case of facioscapulohumeral dystrophy (FSHD) presenting for emergency appendectomy. Preoperative investigations and lung function were adequate. The patient underwent general anesthesia with propofol, cisatarcurium, and remifentanil and was maintained on total IV anesthesia for the duration of surgery. Continuous neuromuscular monitoring was carried out at two sites and the patient responded normally to intubating dose of cisatracurium and subsequent top-up doses, showing no increased sensitivity or need for dose reduction. The patient was hemodynamically stable with propofol and remifentanil infusions, with inhalational agents purposefully avoided. The patient was reversed with anticholinesterase and good train-of-four (TOF) ratio (>90%) was ensured before being shifted to recovery. The patient had a further uneventful course in the hospital.

## Introduction

Facioscapulohumeral dystrophy (FSHD) is a genetic muscular dystrophy characterized by progressive weakness involving the facial, scapular and upper-arm muscles, with relative sparing of the pelvic and lower limb muscles except for dorsiflexors of foot [1,2]. Symptoms of FSHD usually start around 20 years of age with a slow progression of the weakness and around 20% of patients require wheelchair support at some time in their lives. Cardiac and respiratory involvement are rare and life expectancy is near normal in most of these patients. Muscular dystrophies are a perennial nightmare of the anesthesiologist, especially during emergency surgeries, as a wide range of complications including rhabdomyolysis, hyperkalemia, associated cardiac comorbidities, and risk of malignant hyperthermia (MH) influence the anesthetic management [3]. We present such a patient who was posted for emergency laparoscopic appendectomy.

## Case presentation

A 33-year-old male patient, a known case of FSHD for 12 years presented to the operation theater in Amrith Hospital, Chennai, for emergency laparoscopic appendectomy. Patient was diagnosed at age of 21 years with FSHD involving facial muscles and weakness of shoulder girdle muscles especially more on the right side. Muscle biopsy of the right deltoid had confirmed the diagnosis then. He was not on any medications. He had difficulty in raising his arm above the shoulder with obvious wasting of the pectorals on inspection. Blood counts were normal except for platelets being 93 X 10^9 ^L. His creatine phospokinase was well within normal limits. Patient did not have any cardiac symptoms and his electrocardiogram was normal. Spirometry done preoperatively showed a forced vital capacity (FVC) of 3.01 L (69% predicted) and a FEV1/VC of 0.92, which was 112% predicted .

He was accepted for surgery under general anesthesia. Regional anesthesia was not considered in view of laparoscopic approach and emergent nature of surgery. Due risks and need for postoperative ventilation, if required, was explained to the patient. The anesthesia machine was prepared in anticipation of MH, even though MH has been reported only in patients with Duchenne muscular dystrophy and Becker's dystrophy. Anesthesia machine was cleared of vaporizers, carbon dioxide-absorbent changed and the system flushed with oxygen for 30 mins at 10 L/min. Patient was sedated with intravenous fentanyl 150 mcg and midazolam 1 mg. Neuromuscular monitoring was established with train-of-four (TOF) responses using TOF-WATCH concurrently in the right ulnar nerve as well as right posterior tibial nerve, and supramaximal stimulus was obtained at 50 Ma (TOF-WATCH gives TOF ratio if all four responses are present if not the number of responses alone). The extent of TOF after pre-induction sedation showed 98% in the right-upper limb, whereas it was 97% in the right popliteal nerve stimulation. He was induced with IV propofol 150 mg and it was decided to give incremental dose of IV cisatracurium to check for sensitivity to non-depolarizing muscle relaxants as done by Nitahara et al. in their case management of FSHD [4]. Cisatracurium 4 mg was administered (at 0.05 mg initially) and the TOF after five minutes showed three twitches in the upper and lower limbs. Additional cisatracurium 7 mg (total dose of 0.15 mg/kg) was administered and the TOF measured after three minutes was 0 response in both the limbs. The patient was intubated and the surgeon proceeded with laparoscopic appendectomy. The anesthesia was maintained with manual total IV anesthesia (TIVA) using propofol 80-120 mcg/kg/min and remifentanil 0.25 mcg/kg/min with oxygen and air mixture. IV morphine 3 mg was administered 40 minutes into surgery as patient developed tachycardia and the blood pressure was rising. The TOF was persistently 0 for 45 minutes of anesthesia, following which patient developed 1 response in the right ulnar nerve, while the right posterior tibial nerve TOF revealed 2 responses. We found the TOF values between the two limbs being discrepant, which couldn’t be attributed to any known factor and decided to go ahead with the higher value from the lower limb and administered a top-up dose of cisatracurium 2 mg. Subsequently, TOF became 0 in both the limbs and another dose of cisatracurium 2 mg was administered 22 minutes later, after TOF in the lower limb revealed 2 responses. Surgery lasted for 79 minutes, at the end of which the patient had spontaneous attempts at breathing and the TOF (leg) revealed 3 responses around 19 minutes after the last dose of cisatracurium. Patient was reversed with neostigmine and glycopyrrolate. After ensuring a TOF ratio above 90%, the patient was extubated and shifted to recovery. The patient's end-tidal carbon dioxide and temperature were well within normal limits throughout the procedure. Patient’s post anesthesia care unit stay was quiet uneventful, with room air saturation being around 98-99% and hemodynamic parameters stable. The patient was shifted to ward onto a monitored bed.

## Discussion

Major anesthesia-related adverse events related to muscular dystrophies have always been associated with Duchenne muscular dystrophy and Becker’s dystrophy [5]. FSHD is the third most common inherited muscular dystrophy after Duchenne and myotonic dystrophies, manifesting itself in the form of shoulder girdle and facial weakness in the second or third decade [5]. It is otherwise described as a benign dystrophy, since life expectancy is not affected much due to sparing of the cardiac and respiratory systems. Muscular dystrophies can cause a multitude of complications including sensitivity to muscle relaxants, rhabdomyolysis, hyperkalemia, MH-like crises, and, rarely, frank MH. Diagnosis and knowledge about the condition prior to anesthesia is said to prevent most of the untoward complications [6] .Very few case reports exist about anesthesia in patients with FSHD. 

In our case, sensitivity of our patient to neuromuscular blocking agent was essentially normal. The choice of muscle relaxant was based on the fact that, even though rocuronium has been used safely in muscular dystrophies, its reversal agent sugammadex has been quoted to have an unpredictable response and cisatracurium is an intermediate-acting agent with an organ-independent elimination [7,8]. Our lack of experience with rocuronium and sugammadex in muscular dystrophies was also a major factor in favoring use of cisatarcurium. We tried administering a smaller initial dose of cisatarcurium for intubation, as done by Nitahara et al. using vecuronium, but the full induction dose of 0.15 mg/kg (3XED95) of cisatracurium was required for achieving intubating conditions [4]. The initial dose lasted for 45 minutes well within the range in normal patients. Even subsequent two top-up doses lasted for 22 and 19 minutes, respectively, both being around of 20 minutes (the normal duration following maintenance dose of 0.03 mg/kg). Even though sevoflurane has been safely used by authors in their case management of a patient with FSHD, we decided to go for TIVA as it has been proven to be safe in neuromuscular disorders [9,10]. Intraoperatively, the hemodynamic parameters were quite stable and temperature ranged between 36.3 and 36.9°C. Neuromuscular monitoring plays a crucial role in intraoperative management of muscular dystrophies. Monitoring the TOF responses at multiple sites has been suggested by authors in view of responses being unpredictable due to non-uniform atrophy of muscles [11]. Consistent with that was our observation of discrepancy in the TOF between the two limbs, as we found lower-limb TOF to be always higher than ulnar nerve. Whether this has any correlation to the disease pattern has to be corroborated further. Even though anti-cholinesterases are supposed to precipitate hyperkalemia in myopathy patients, we reversed the patient with neostigmine and glycopyrrolate, as there were no literature suggesting such an complication in a benign dystrophy like FSHD [3]. In short, the patient had an uneventful anesthesia course with a normal response to cisatracurium. Trevisan et al. mention that most of all anesthesia-related complications occur in undiagnosed muscular dystrophies [6]. Since we had knowledge of the patient's myopathy and adequate time to evaluate the patient prior to the surgery with respect to the respiratory and cardiac capacity, we feel we were able to proceed with the procedure, without major adverse anesthesia event.

## Conclusions

Muscular dystrophies pose a huge anesthetic challenge. Adequate preoperative preparation and choice of anesthetic agents would help minimize the adverse complications. Increasing positive evidence of use of safer agents like rocuronium and sugammadex, combined with appropriate neuromuscular monitoring could make anesthesia absolutely safe in patients with muscular dystrophies.
